# Annotations

**Published:** 1908-03-28

**Authors:** 


### Annotations.

### The Rehabilitation of the Oyster.

Sand is steadily replacing sewage in the environment of the modern oyster bed; so says the annual report of the Oyster Merchants' and Planters' Association. The prevention of pollution of waterways and estuaries has absorbed the energies of the Association with the most beneficial results, for the oyster has now lost all virtue as a subject for silly season scares. It is many years since any outbreak of zymotic disease has been traced to these creatures, and the Association, together with the Fishmongers' Company, is to be congratulated on the success with which it has grappled with a very serious question. It is not too much to say that the oyster trade would to-day have been non-existent if prompt measures to restore public confidence had been neglected. The rehabilitation of the oyster has been effected with but little public advertisement, and has therefore, perhaps, escaped its due measure of popular appreciation; but it is none the less a fact, and one for which the oyster-eating public should be grateful to the two corporations concerned. To those who remember the suspicion with which everyone of these delicacies was regarded not many years ago, the change that has been brought about so quietly is indeed an extraordinary one, and it is the more satisfactory that private enterprise has reached this result without any form of State intervention. For food which is of necessity swallowed, without even mastication, in the raw state should be as free from suspicion of bacterial contamination as drinking-water. The conference shortly to be held between the Main Drainage Committee of the London County Council and the Port of London Sanitary Committee on this very question of bacterial aspect of London sewage should be productive of still greater security in this respect than that which oyster consumers seem to be already enjoying.

### "The Psychology of Woman."

If Dr. Claye Shaw probes deeply into the mind of woman, he does so with the gentlest of hands. With an air of delicate courtesy he traces back her mental processes to the needs and instincts of primitive existence, analyses her emotions into their crudest elements, and gazes with critical eyes into her psychological future. His recent lecture at the Institute of Hygiene on "The Psychology of Woman" was deeply interesting and suggestive, enlivened throughout with simple illustrations drawn from everyday life, and dry touches of an almost sardonic humour. Original reflections upon this most elusive subject lead him to the conclusion that man's boasted superiority is a myth. Up to the age of puberty the mental processes of boys and girls are practically identical: beyond this point they diverge. But this divergence, according to Dr. Claye Shaw, is not in the direction of superiority for man and inferiority for woman. The march of civilisation has emphasised and elaborated the natural differences between the sexes, but at no time has woman been psychologically the inferior: her environment has been against her, but she has never succumbed to it. Dr. Claye Shaw states, on the authority of Professor Marshall, and contrary to the usual belief, that, height for height, man and woman show no appreciable difference in weight of brain: what man gains in the upper parts, woman makes up for in the basal ganglia. In memory and ideation, again, there seems to be but little to choose between the sexes; while in perception and emotions woman is by far the more richly endowed. Here we may note that Dr. Claye Shaw has a friendly word of ingenious defence for those much-abused women, the butterfly and the flirt. He agrees with the view that the woman's mind is the complement of the man's: her psychology is incomplete until she is happily married. But he asserts that, although her mind works harmoniously with her husband's in the life of the family, the conduct of public affairs must ever rest in the hands of one sex alone. Whether this principle can be upheld or not, it is one which might justifiably engage the thoughts of the more militant of woman suffragists. If Dr. Claye Shaw is right, the question which these ladies should ask themselves is not, "Do we wish to share the reins of Government with man?" but "Do we wish to depose him altogether from the box-seat?"

### Liability of Anæsthetists.

Elsewhere (p. 689) will be found a summary of the important discussion on this subject at the Medico-Legal Society's meeting. Mr. Henslowe Wellington's analysis of the situation leads him to the conclusion that the administration of any anæsthetic, local or general, by an unqualified person should be made a penal offence. We fear it will be some time before Parliament or the Privy Council will consent to any such measure of protection for the public, for the prohibition of unqualified practice in general must logically follow. Certainly, however, it does not seem too much to expect that the control of general anæsthesia should be confined to qualified practitioners. We should like to see the appointment of a commission to inquire into the administration of anæsthetics and the relations which exist between the anæsthetist and the surgeon. As matters stand, there is an occasional tendency on the part of surgeons to prolong operations in hospital unduly, because it is the anæsthetist who will be requested to attend a subsequent inquest. The position and responsibility of the anæsthetist are thus frequently anomalous, and the results to patients are not always what they ought to be. Another point is one briefly mentioned by Mr. Wellington, and illustrates a reverse tendency. That is the impatience of a busy surgeon with appointments to keep outside his hospital, whereby pressure is sometimes brought to bear on the anæsthetist to hurry the induction more than is consistent with safety. The thanks of the profession are due to the Medico-Legal Society for initiating the discussion on these important points.

